# Risk factors associated with 31-day unplanned readmission in 50,912 discharged patients after stroke in China

**DOI:** 10.1186/s12883-018-1209-y

**Published:** 2018-12-26

**Authors:** Tiancai Wen, Baoyan Liu, Xia Wan, Xiaoping Zhang, Jin Zhang, Xuezhong Zhou, Alexander Y. L. Lau, Yanning Zhang

**Affiliations:** 1 School of Computer Science, Northwestern Polytechnical University Xi’an, Shangxi Province, 710129 China; 2 Institute of Basic Research in Clinical Medicine, China Academy of Chinese Medical Sciences, Beijing, 100700 China; 3 Data Center of Traditional Chinese Medicine, China Academy of Chinese Medical Sciences, Beijing, 100700 China; 4 Institute of Basic Medical Sciences at Chinese Academy of Medical Sciences / School of Basic Medicine at Peking Union Medical College, Beijing, 100005 China; 5 School of Computer and Information Technology, Beijing Jiaotong University, Beijing, 100044 China; 6 Faculty of Medicine, The Chinese University of Hong Kong, Hong Kong, China

**Keywords:** Stroke, Unplanned readmission, Hospitalization, Cover sheet of medical record, Risk factor

## Abstract

**Background:**

Unplanned readmission within 31 days of discharge after stroke is a useful indicator for monitoring quality of hospital care. We evaluated the risk factors associated with 31-day unplanned readmission of stroke patients in China.

**Methods:**

We identified 50,912 patients from 375 hospitals in 29 provinces, municipalities or autonomous districts across China who experienced an unplanned readmission after stroke between 2015 and 2016, and extracted data from the inpatients’ cover sheet data from the Medical Record Monitoring Database. Patients were grouped into readmission within 31 days or beyond for analysis. Chi-squared test was used to analyze demographic information, health system and clinical process-related factors according to the data type. Multilevel logistic modeling was used to examine the effects of patient (level 1) and hospital (level 2) characteristics on an unplanned readmission ≤31 days.

**Results:**

Among 50,912 patients, 14,664 (28.8%) were readmitted within 31 days after discharge. The commonest cause of readmissions were recurrent stroke (34.8%), hypertension (22.94%), cardio/cerebrovascular disease (13.26%) and diabetes/diabetic complications (7.34%). Higher risks of unplanned readmissions were associated with diabetes (OR = 1.089, *P* = 0.001), use of clinical pathways (OR = 1.174, *P* < 0.001), and being discharged without doctor’s advice (OR = 1.485, *P* < 0.001). Lower risks were associated with basic medical insurances (OR ranging from 0.225 to 0.716, P < 0.001) and commercial medical insurance (OR = 0.636, *P* = 0.021), compared to self-paying for medical services. And patients aged 50 years old and above (OR ranging from 0.650 to 0.985, *P* < 0.05), with haemorrhagic stroke (OR = 0.467, P < 0.001), with length of stay more than 7 days in hospital (OR ranging from 0.082 to 0.566, P < 0.001), also had lower risks.

**Conclusions:**

Age, type of stroke, medical insurance status, type of discharge, use of clinical pathways, length of hospital stay and comorbidities were the most influential factors for readmission within 31 days.

## Background

Stroke is an important public health concern in China as the leading cause of morbidity and mortality [1]. In *the Report on Cardiovascular Diseases in China (2017)*, it was estimated that approximately 13 million Chinese people had suffered a stroke [2]. The mortality rate of stroke in China has been estimated to be 4 to 5-fold greater than in western developed countries [3]. Meanwhile, stroke causes an enormous economic burden in China; the hospitalization expenses for intracranial hemorrhage and cerebral infarction have increased by approximately 19 and 25% annually since 2004 [4, 5].

Readmissions after hospitalization increases the burden on patients and is a waste of health resources [6]. Unplanned early readmission is a potentially useful indicator for monitoring the quality of hospital care [7]; The National Health Commission of China uses unplanned readmission within 31 days of discharge for the same disease as an indicator of the medical quality of hospitals [8], including stroke [9]. Unplanned readmission within 31 days of discharge among patients with stroke may reflect unresolved problems at discharge or a lack of effective outreach care after discharge [10]. It has been shown that the risk of readmission can be reduced by better care during the index hospitalization, improvements in discharge planning and follow-up care/outreach health care, or better transitions between inpatient and outpatient healthcare teams [11]. In addition, patients with stroke who were readmitted within one month after discharge had a worse functional outcome or higher mortality and medical costs than those who were not readmitted [12–14]. Therefore, in order to prevent avoidable readmissions, it is important to identify risk factors associated with 31-day unplanned readmission in discharged patients after stroke.

The most common causes of readmission among patients with stroke include recurrent stroke, infections, and cardiac diseases [15]. In addition, social and financial factors may contribute to readmissions in patients discharged after stroke [16]. Although several clinical trials and observational studies have examined the causes of readmission within one month of discharge after stroke, the impact of social, financial, hospital or health system-related factors remain uncertain due to small sample sizes or limited patient cohorts [10, 17]. In China, the cover sheet is the first page of the medical record for each individual inpatient, which provides detailed information, including patient characteristics, all diagnoses, clinical treatments or procedures, social circumstances, financial status, hospital type, cost and type of payment [18, 19]. For patients with consecutive medical records, information about their past histories and all treatments provided after stroke are also recorded and linked by an unique individual identifier [20].

The aim of this study was to determine the factors associated with 31-day unplanned readmission in patients discharged after stroke by extracting the data of the index hospitalization and subsequent readmission from a large database of the medical record cover sheet.

## Methods

### Data source

This study used data collected from the Cover Sheet of Stroke Inpatients’ Medical Records from the Monitoring Database of National Key Traditional Chinese Medicine (TCM) Project, which includes data from more than 50 million cover sheets from 578 TCM hospitals or TCM departments in general hospitals across China from Jan 2015 to Dec 2016. Diagnosis of TCM means patients are diagnosed by TCM theory (syndrome differentiation) with whole body, pulse and tongue symptoms. Treatment of TCM means patients were treated by Chinese herb or acupuncture. Nowadays, combined diagnosis and treatment of TCM and Western Medicine (WM) are the most common strategy used in TCM hospitals or clinics. Therefore, in the TCM hospitals or TCM departments, patients are diagnosed by both WM and TCM theory, then based on the western stroke treatment, doctors may add Chinese herb or acupuncture, depending on the severity of stroke patients. And in the tertiary TCM hospital, department of internal neurology or stroke unit is used for stroke patients.

The criteria for data inclusion in this study were 1) patients aged between 18 and 100 years with a principal diagnosis of haemorrhagic stroke (ICD-10 code: I61) or ischemic stroke (ICD-10 code: I63) (forty-one patients coded I64 were not considered because of the small sample size.); 2) patients readmitted to any hospital after discharge due to a stroke-related condition. The first onset of stroke was taken as the index event and readmission as the endpoint event. We were unable to evaluate the outcomes of patients who did not return to a hospital; they may have fully recovered or they may have died. Therefore, patients without readmission information, or with planned readmission ≤31 days, or readmitted not for a stroke-related condition (such as toxic effect of snake venom or foodborne *Bacillus cereus* intoxication) were excluded. In order to protect patients’ privacy, all data were anonymized by removing sensitive personal information, and subjects were identified by a hospital number or a personal identification number managed by the database administrators.

### Study variables

Before undertaking the data analysis, occupation and the level of hospital were re-classified: ‘Manager and staff’ referred to civil officers, specialized technicians, clerks and leaders of organizations; ‘Self-employed’ included freelancers. In China, the retirement age for females and males was 55 and 60 years old, respectively [21]; and most Chinese reported that they had no job after retirement, so those people aged 55 (for females) or 60 (for males) years old and above without any job were classified to the ‘retiree’ group. Primary and secondary hospitals were combined, so the level of hospital classified into ‘tertiary hospital’ and ‘primary or secondary hospital’.

Data included basic hospital information (including the level of hospital and type of hospital), patient demographic information (including sex, age, principal discharge diagnosis, and comorbidities), health care services and clinical processes provided (including admission route, medical expense payment, and use of TCM diagnosis/treatment). Due to the large data set, a great number of comorbidities were identified from the database. Based on the literature review [22–24], we pre-specified ten comorbidities that were regarded as contributing diseases to stroke, namely, hypertension, diabetes, dyslipidemia, and heart disease (including coronary heart disease, angina pectoris, arrhythmia, heart failure, atrial fibrillation, and myocardial infarction).

### Statistical analysis

The data analysis and map drawing were performed by using R (version 3.4.3). Potential risk factors for readmission were compared by univariable analysis between patients readmitted ≤31 days and > 31 days with the chi-squared test. The multilevel logistic modeling (MLM) (R package, lme4 version 1.1–15) was used to examine the effect of patient (level 1) and hospital (level 2) characteristics on an unplanned readmission ≤31 days. The variables with a *P* value of < 0.1 from the univariable analysis were entered into the MLM.

Two steps were conducted to build the MLM: (1) an empty model containing no independent variables was used to describe the variation between hospitals; (2) all patient and hospital-level variables were entered into a full model to study fixed effects. In the final model, *P* values of < 0.05 were considered significant. Adjusted odds ratios (OR) with 95% confidence interval (CI) were calculated. The degree of heterogeneity of clinical services from the same hospital was computed using the interclass correlation.

## Results

**Fig. 1:**
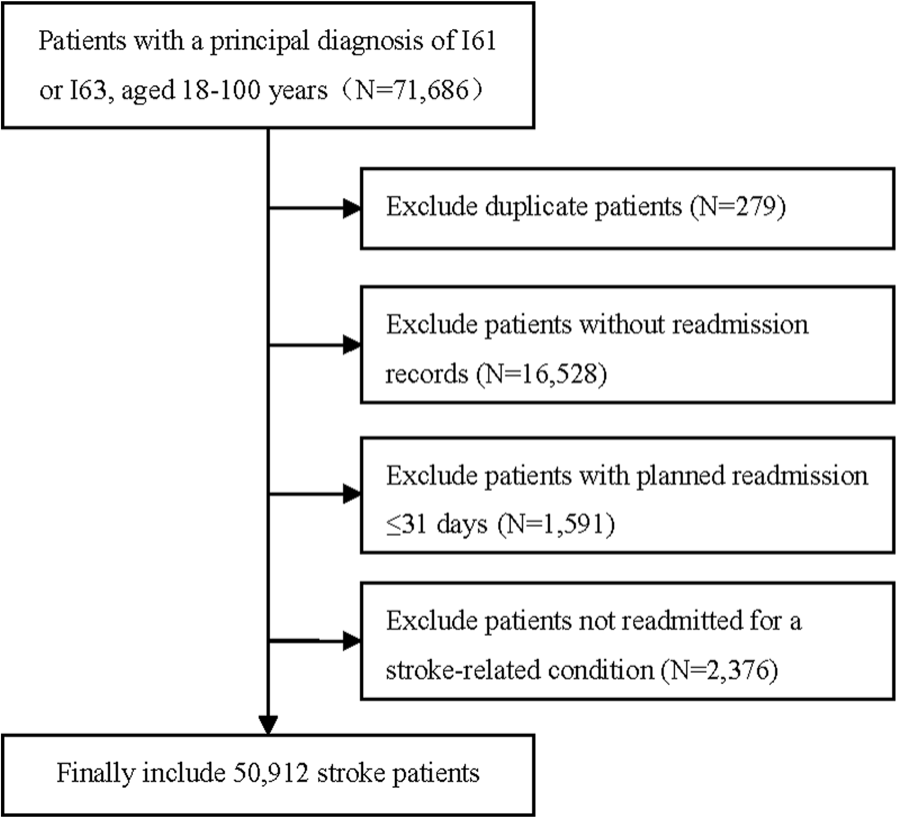
Flowchart for patient selection

**Fig. 2:**
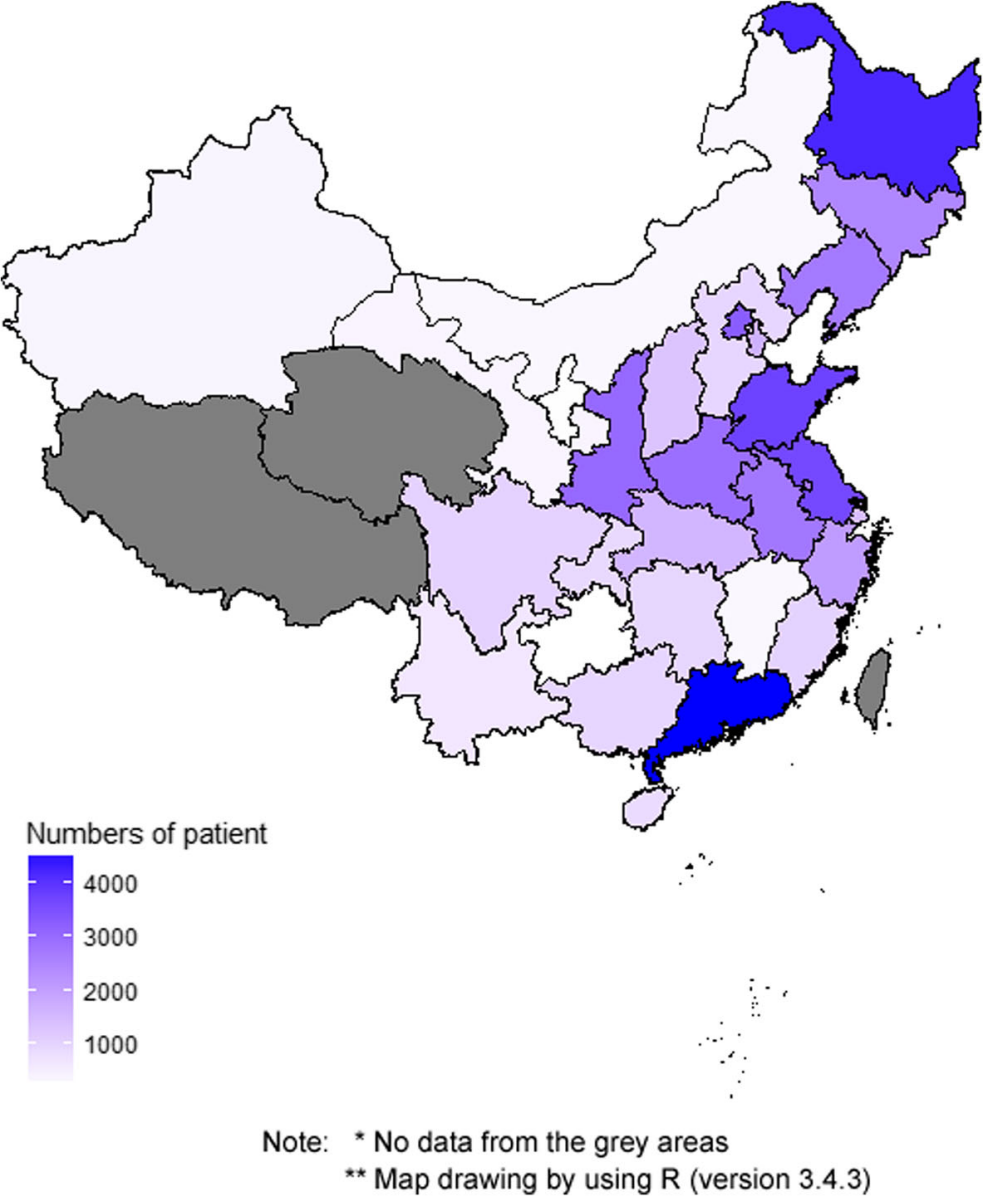
The distribution of included patients across China. Number of patients

The results of the data inclusion procedure are shown in Fig. 1. A total of 50,912 eligible patients with stroke from 375 hospitals distributed among 29 provinces, municipalities or autonomous districts across China, who experienced an unplanned readmission after stroke from 2015 to 2016, were included in the study (Fig. 2).

**Fig. 3:**
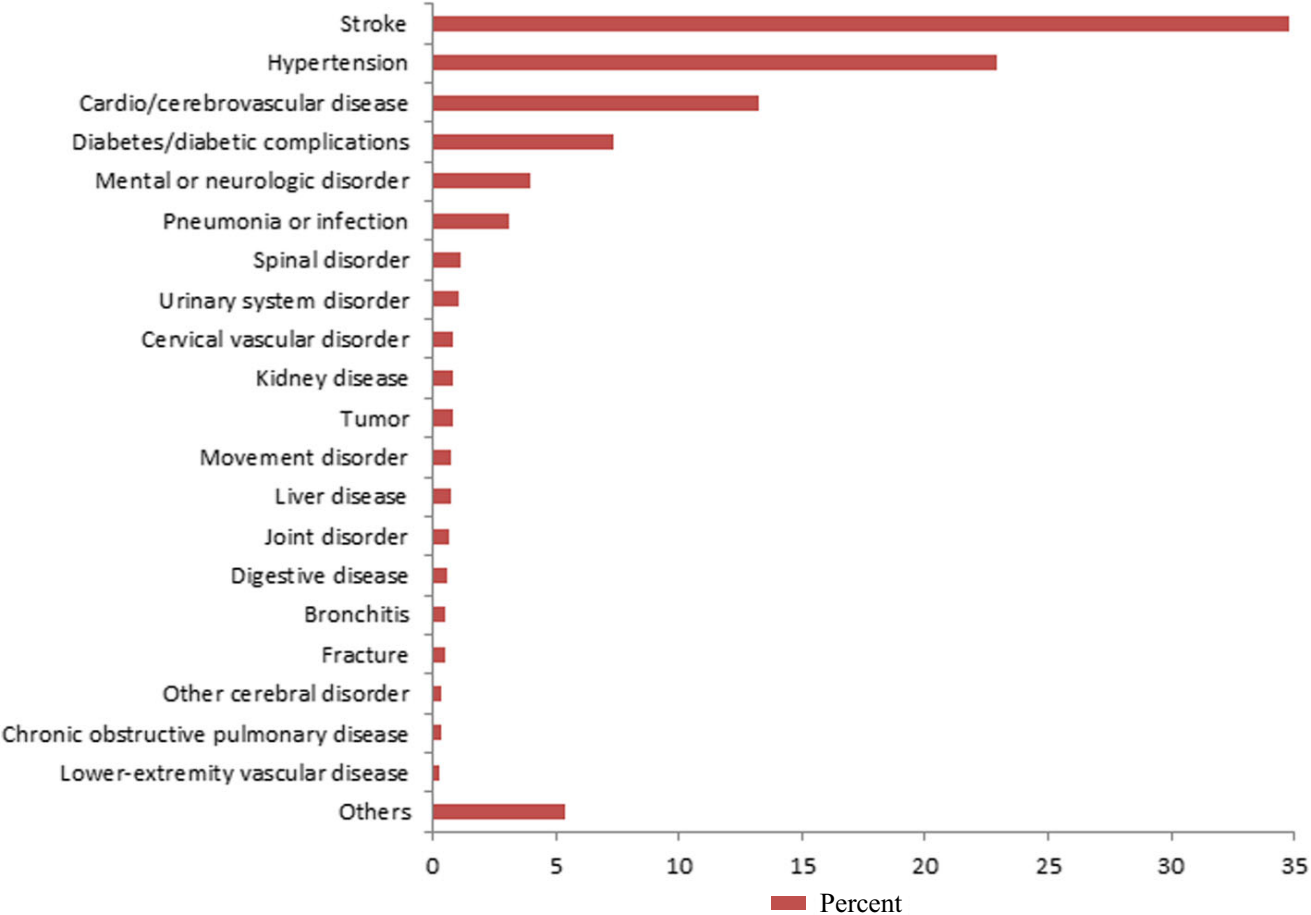
Reasons for readmissions. Percent

Among all patients, 28.8% (14,664) were readmitted ≤31 days after discharge. The reasons for readmissions are summarized in Fig. 3. The commonest causes were recurrent stroke, hypertension, cardio/cerebrovascular disease and diabetes/diabetic complications.

Most of the patients were patients with ischemic stroke, males, retirees/unemployed, married, covered by the Urban Employees’ Basic Medical Insurance and with admission route from an outpatient service. More than 93% of subjects were collected from tertiary hospitals, especially from TCM hospitals with TCM treatment. The proportion of hospitals using clinical pathways was 69.6%, but the proportion for patients was just 30.2%. Most of the variables were statistically different between patients readmitted ≤31 days and > 31 days after discharge, except three variables (comorbidities with dyslipidemia or heart disease, and surgical treatment). (Table 1).

**Table 1:** Patient characteristics, comorbidities, health system and clinical process-related factors for the risk of hospital readmission ≤31 days

Factor	Readmitted ≤ 31 days (*N* = 14,664)	Readmitted>31 days (*N* = 36,248)	Total (*N* = 50,912)	*P*-Value
	*n*(%)	*n*(%)	*n*(%)	
Patient characteristics				
Principal discharge diagnosis				< 0.001
I61Haemorrhagic stroke	3144(21.4)	4097(11.3)	7241(14.2)	
I63 Ischemic stroke	11,520(78.6)	32,151(88.7)	43,671(85.8)	
Sex - women	5392(36.8)	13,842(38.2)	19,234(37.8)	0.003
Age (years)				< 0.001
18–49	1545(10.5)	2625(7.2)	4170(8.2)	
50–59	2837(19.4)	6191(17.1)	9028(17.7)	
60–69	3911(26.7)	10,384(28.7)	14,295(28.1)	
70–79	3735(25.5)	10,519(29.0)	14,254(28.0)	
80 and above	2636(18.0)	6529(18.0)	9165(18.0)	
Occupation				< 0.001
Manager and staff	1260(8.6)	2621(7.2)	3881(7.6)	
Manufacturing worker	920(6.3)	3230(8.9)	4150(8.2)	
Farmer	1571(10.7)	4577(12.6)	6148(12.1)	
Self-employed	308(2.1)	571(1.6)	879(1.7)	
Retiree	8655(59.0)	21,544(59.4)	30,199(59.3)	
Others	1950(13.3)	3705(10.2)	5655(11.1)	
Marital status				0.005
Single / widow/ divorce/other	1674(11.4)	4465(12.3)	6139(12.1)	
Married	12,990(88.6)	31,783(87.7)	44,773(87.9)	
Medical expense payers				< 0.001
Self-paying medical service	2121(14.5)	3121(8.6)	5242(10.3)	
Urban employee basic medical insurance	8750(59.7)	22,915(63.2)	31,665(62.2)	
Urban resident basic medical insurance	1547(10.6)	4400(12.1)	5947(11.7)	
New rural cooperative medical scheme	1598(10.9)	4250(11.7)	5848(11.5)	
Medical financial assistance fund for the poor	64(0.4)	230(0.6)	294(0.6)	
Commercial medical insurance	93(0.6)	268(0.7)	361(0.7)	
Free medical service	390(2.7)	718(2.0)	1108(2.2)	
Other social insurance	101(0.7)	346(0.95)	447(0.9)	
Comorbidities				
Hypertension - yes	9339(63.7)	22,200(61.2)	31,539(62.0)	< 0.001
Diabetes - yes	4087(27.9)	9568(26.4)	13,655(26.9)	0.001
Dyslipidemia - yes	2003(13.7)	5079(14.0)	7082(13.9)	0.298
Heart disease - yes*	4373(29.8)	10,889(30.0)	15,262(30.0)	0.625
Health system and clinical processes				
Level of hospital -tertiary hospital	13,944(95.1)	33,689(93.0)	47,633(93. 6)	< 0.001
Type of hospital				< 0.001
Western medicine (WM) hospital	106(0.8)	409(1.1)	515(1.0)	
TCM hospital	13,491(92.0)	32,793(90.5)	46,284(90.9)	
TCM-WM hospital	976(6.7)	2826(7.8)	3802(7.5)	
Ethnic minority hospital	91(0.6)	220(0.6)	311(0.6)	
Admission route				< 0.001
Emergency	4592(31.3)	11,937(33.0)	16,529(32.5)	
Outpatient	9541(65.1)	22,420(61.9)	31,961(62.8)	
Others (such as Transferred from other hospitals)	531(3.6)	1891(5.2)	2422(4.8)	
Length of hospital stay (days)				< 0.001
1–7	2094(14.3)	2531(7.0)	4625(9.1)	
8–14	5432(37.0)	14,163(39.1)	19,595(38.5)	
15–21	4387(29.9)	10,250(28.3)	14,637(28.8)	
22–28	1982(13.5)	3699(10.2)	5681(11.2)	
29 and above	769(5.2)	5605(15.5)	6374(12.5)	
Use of clinical pathways** - yes	4707(32.1)	10,664(29.4)	15,371(30.2)	< 0.001
Surgical treatment - yes	705(4.8)	1794(5.0)	2499(4.9)	0.503
Use TCM treatment - yes	13,224(90.2)	31,560(87.1)	44,784(88.0)	< 0.001
Type of discharge				0.033
Leave or transfer with doctor’s advice	14,100(96.2)	34,994(96.5)	49,094(96.4)	
Others***	564(3.8)	1254(3.5)	1818(3.6)	

*Heart disease includes coronary heart disease, angina pectoris, arrhythmia, heart failure, atrial fibrillation, and myocardial infarction

**In terms of hospitals, 69.6% of hospitals used clinical pathways

***Others include patients discharged without doctor’s advice and those deceased

**Fig. 4:**
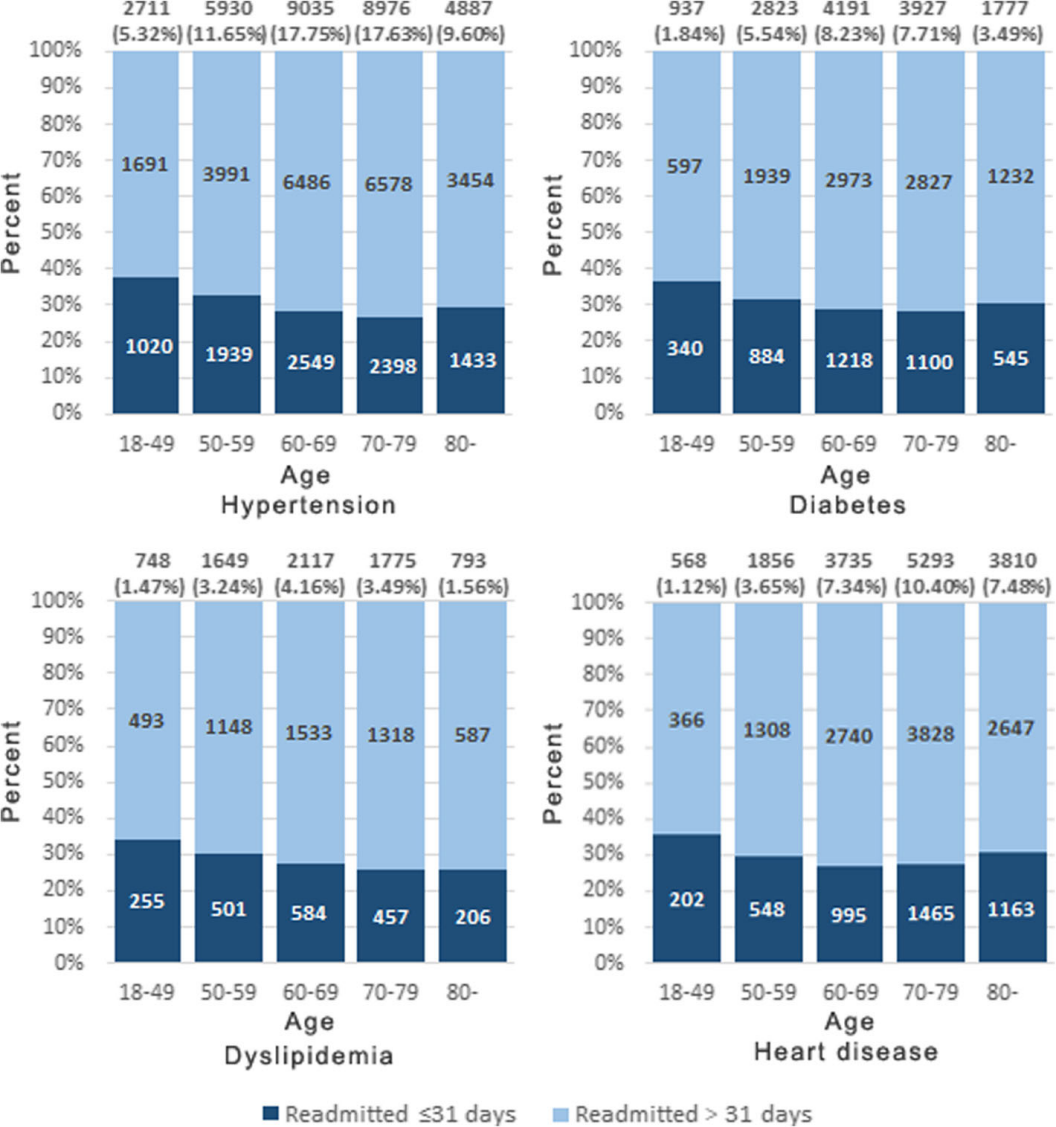
The proportions for patients readmitted ≤31 days after discharge due to a stroke-related condition in different age groups with different diseases. Readmitted **≤**31 days. Readmitted > 31 days

Among all the patients, the proportion of patients with hypertension was 5.32% in the 18–49 age group, which was the lowest one among all the age groups. However, among those patients with hypertension, the proportion for patients readmitted ≤31 days after discharge due to a stroke-related condition in the 18–49 age group was highest (38.7%), compared to other age groups. The situations for diabetes, dyslipidemia, heart diseases were the same as hypertension. The proportions for patients readmitted ≤31 days after discharge in the 18–49 age group were also highest among patients with the above three diseases, 36.9, 35.5 and 35.8%, respectively. (Fig. 4).

The MLM analyses showed that patients with diabetes (OR = 1.089, *P* = 0.001), using clinical pathways (OR = 1.174, *P* < 0.001), and leaving or transferred without the doctor’s advice (OR = 1.485, *P* < 0.001) had a higher risk of unplanned readmission ≤31 days. Whereas, compared to patients with self-paying medical service, patients with basic medical insurances (OR = 0.504, P < 0.001), urban resident basic medical insurance (OR = 0.514, P < 0.001), new rural cooperative medical scheme (OR = 0.643, P < 0.001), medical financial assistance fund for the poor (OR = 0.309, P < 0.001)) and commercial medical insurance (OR = 0.636, *P* = 0.021) were at a lower risk. A lower risk was also observed in patients aged 50 years old and above (OR ranging from 0.650 to 0.985, *P* < 0.05), with haemorrhagic stroke (OR = 0.467, P < 0.001), or if length of stay was more than 7 days in hospital (OR ranging from 0.082 to 0.566, P < 0.001) (Table 2).

**Table 2:** Multilevel logistic regression, factors associated with readmission ≤31 days^a^

Factor	OR	95% CI		*P*-Value
		Lower	Upper	
Patient characteristics				
Principal discharge diagnosis - I61 Haemorrhagic stroke	1			
I63 Ischemic stroke	0.467	0.437	0.498	< 0.001
Sex – male	1			
female	0.995	0.950	1.042	0.842
Age (years) - 18-49	1			
50–59	0.820	0.749	0.898	< 0.001
60–69	0.737	0.662	0.819	< 0.001
70–79	0.725	0.650	0.808	< 0.001
80 and above	0.879	0.784	0.985	0.027
Occupation - Manager and staff	1			
Manufacturing worker	0.962	0.846	1.094	0.556
Farmer	1.090	0.959	1.238	0.187
Self-employed	0.958	0.785	1.169	0.674
Retiree	1.092	0.984	1.212	0.099
Others	1.072	0.961	1.195	0.211
Marital status –Single / widow/ divorce/other	1			
Married	0.969	0.898	1.046	0.422
Medical expense payers - Self-paying medical service	1			
Urban employee basic medical insurance	0.504	0.467	0.544	< 0.001
Urban resident basic medical insurance	0.514	0.466	0.567	< 0.001
New rural cooperative medical scheme	0.643	0.578	0.716	< 0.001
Medical financial assistance fund for the poor	0.309	0.225	0.424	< 0.001
Commercial medical insurance	0.636	0.433	0.934	0.021
Free medical service	0.865	0.735	1.017	0.079
Other social insurance	0.447	0.345	0.581	< 0.001
Comorbidities				
Hypertension - no	1			
yes	0.966	0.918	1.017	0.188
Diabetes - no	1			
yes	1.089	1.035	1.145	0.001
Health system and clinical processes				
Rank of hospital -primary and secondary hospital	1			
tertiary hospital	1.168	0.845	1.614	0.348
Type of hospital - Western medicine (WM) hospital	1			
TCM hospital	1.194	0.778	1.833	0.417
TCM-WM hospital	1.287	0.768	2.156	0.339
Ethnic minority hospital	1.360	0.564	3.279	0.493
Admission route - Emergency	1			
Outpatient	0.994	0.929	1.064	0.860
Others (such as Transferred from other hospitals)	1.140	0.945	1.374	0.170
Length of hospital stay (days) - 1-7	1			
8–14	0.444	0.411	0.478	< 0.001
15–21	0.459	0.424	0.496	< 0.001
22–28	0.516	0.470	0.566	< 0.001
29 and above	0.092	0.082	0.103	< 0.001
Use of clinical pathways - no	1			
yes	1.174	1.108	1.243	< 0.001
Use TCM treatment - no	1			
yes	1.036	0.944	1.136	0.458
Type of discharge - Leaving or transferred with doctor’s advice	1			
Others*	1.485	1.272	1.733	< 0.001

*Others include patients discharged without doctor’s advice and those deceased

^a^Random-effects variance:0.792; interclass correlation: 0.194; OR: odds ratio; CI: confidence interval

## Discussion

This study comprehensively assessed the impact of both disease-related and unrelated factors on the risk of 31-day unplanned readmission after stroke, including social, financial, hospital or health system-related factors. We found that recurrent stroke was the most important reason for readmission. Age, type of stroke, medical expense payment, type of discharge, use of clinical pathways, length of hospital-stay and some comorbidities were the most influential factors for readmission within 31 days for a stroke related condition.

Patients aged younger than 50 years old had a higher readmission risk than older patients. It is probably because of large increases in the common stroke risk factors in that age group, such as tobacco use, hypertension, and diabetes mellitus [25–27]. In China, the 2010 Global Adults Tobacco Survey results showed that from 2002 to 2010, the male current smoking prevalence in the age group of 40–59 increased dramatically by nearly 70%, while current smoking prevalence among other age groups didn’t change significantly [28]. The hypertension prevalence increased among adults aged 35 years old and above. According to China Kadoorie Biobank Study, compared with other age groups, the hypertension prevalence was 12.6% in the 35–39 age group, also with a greater proportion of uncontrolled hypertension [29]. The prevalence of diabetes was 3.2 and 11.5% among persons aged 20–39 and 40–59, respectively [30]. In the 18–39 age group, the rate of diabetes awareness was extremely low (5%), also with the lowest treatment and control rate among all the adults aged 18 years old and above [31]. Therefore, it is important to control these potential risk factors, especially for people aged under 50 years.

Patients with haemorrhagic stroke had a higher risk of readmission ≤31 days perhaps because strokes are generally more severe in patients with haemorrhagic stroke. In one study conducted in Denmark, stroke severity was found to be correlated with the probability of having haemorrhagic stroke (2% in patients with the mildest stroke and 30% in those with the most severe strokes) [32].

Use of clinical pathways was also found to be associated with readmission ≤31 days. In order to improve professional practice and treatment outcome, as well as reduce length of stay and hospital cost, National Health Commission of China issued a Guideline for Clinical Pathways Management (pilot version) in 2009 [33], which requires different health organizations/hospitals at each level to make their own clinical pathways for common diseases. This guideline was updated in August 2017, which requires those patients with confirmed diagnosis, but without severe complications, could get clinical pathways. Once those patients under the clinical pathways have severe complications who have to be changed treatment strategy / clinical department, they should quit clinical pathways [34]. Since 2009, most hospitals have developed their clinical pathways for stroke. In our study, 69.6% of hospitals use clinical pathways. But the use of clinical pathways in stroke care is questionable, because the evidence on effectiveness is still inconclusive, not only in China [35], but also in other countries [36]. Thus, further research is needed before widespread implementation of these clinical pathways. However, it should be noted that in the univariable analysis, the prevalence of using clinical pathways in two groups (readmitted ≤31 days and > 31 days) was just 3% difference, which means that this significant difference may be associated with the large sample size and may not be clinically relevant..

Patients with more than 7 days of hospital stay had a lower risk for readmission within 1 month, compared to those with ≤7 days of hospital stay. An Australian study showed that the median length of stay for readmission was 6 days (Q_1_-Q_3_, 2–12) [10]. This indicates that, at least for some stroke patients, 6–7 days of treatment in hospital may be inadequate; and for those patients in serious condition and less than 7 days of hospital stay, transitions to other healthcare services after discharge to prevent unplanned readmission should be considered [37].

Because the data for this study was obtained from the Monitoring Database of National Key TCM Project, there were some limitations in our study. Firstly, more than 91% of patients included in our analysis were from TCM hospitals. Hospitals in China are classified into western medicine (WM) hospitals and TCM hospitals. In almost every county and city, there is at least one TCM hospital. In 2013, there were 3105 TCM hospitals in China, accounting for 12.6% of all hospitals [38]. Our current study included 375 hospitals, covering almost all provinces of China, which might be representative of all TCM hospitals. Nowadays, combined diagnosis and treatment of TCM and WM are the most common strategy used in TCM hospitals or clinics. In China, the basic treatment strategies in the TCM hospitals were the same as those in WM hospitals because all hospitals should follow the Guideline for Stroke Diagnosis and Treatment issued by Chinese Medical Association [39]. In addition, TCM hospitals or clinics should also comply with the Guideline for Stroke Diagnosis and Treatment by TCM issued by State Administration of TCM [40], which means that doctors may add some TCM treatment modalities, such as Chinese herb or acupuncture, depending on the severity of stroke patients. It is unknown whether or not this had an impact on our findings.

Secondly, because these data were secondary data from each inpatient’s cover sheet of medical records and not designed for research purposes, some important medical information was not recorded. For example, data on the severity of stroke could not be extracted from the database. An Australian study reported that being dependent before the initial admission (modified Rankin Scale, 2–5) was significantly associated with readmission within 28 days [10]. Also, the clinical criteria for patient discharge could not be collected from the cover sheet of medical records. Thirdly, we were unable to track patients who did not come back to hospital; they may have fully recovered or they may have died.

Lastly, our study findings using multilevel logistic modeling may face a risk of bias. This is because the odds ratio (OR) is approximately the same as the relative risk (RR) if the outcome of interest is rare [41]. However, in our study the readmission rate was 28.8%. Therefore, we had tried to use the multilevel log-binomial regression. Unfortunately, the results showed that the log-binomial model was inappropriate for our data which may be caused by an imbalance in the numbers of different types of hospitals. Hence, we reported the result of multilevel logistic regression in this article. These limitations may potentially cause some biases in our results. Generalizability to other countries may also be limited because most hospitals were TCM.

## Conclusions

In summary, recurrent stroke, hypertension, cardio/cerebrovascular disease and diabetes/diabetic complications are the most common reasons for readmissions. Age, type of stroke, medical expense payment, type of discharge, use of clinical pathways, length of hospital-stay and some comorbidities were the most influential factors for readmission within 31 days. Interventions aimed at preventing 31-day readmissions should place more attention to patients with comorbidities, or unhealthy behaviors, especially for young and middle-aged patients. Further research is needed before widespread implementation of the clinical pathways for stroke. Hospitals should consider whether patients with ≤7 days of hospital stay may benefit from staying longer.

## Abbreviations

CI: Confidence interval
MLM: Multilevel logistic modeling
OR: Odds ratio
RR: Risk ratio
TCM: Traditional Chinese Medicine
WM: Western medicine
